# Remineralization Effect of Zamzam Water on Initial Artificial Carious Lesion of Permanent Teeth

**DOI:** 10.7759/cureus.32823

**Published:** 2022-12-22

**Authors:** Tamim S Alkhalifah, Luba A Almuhaish, Prabu M Syed Mahin

**Affiliations:** 1 Department of Dentistry, Qassim University, Qassim, SAU; 2 College of Dentistry, Imam Abdulrahman Bin Faisal University, Dammam, SAU; 3 Department of Restorative Dental Sciences, Qassim University, Qassim, SAU

**Keywords:** sodium fluoride, microhardness, demineralization, remineralization, zamzam water

## Abstract

Introduction: Chemical testing showed that Zamzam water is completely safe to drink and has health benefits due to its high percentage of sodium, calcium, magnesium, and many other minerals. The purpose of this study was to evaluate the remineralization effect of Zamzam water on extracted premolars using the Vickers Microhardness test.

Methods: Teeth samples (N=40) with artificially induced carious lesions were divided randomly into four groups: Study group (I) treated with agitated Zamzam water (n=10), study group (II) treated with non-agitated Zamzam water (n=10), control positive group (III) treated with sodium fluoride (n=10), and control negative group (IV) treated with deionized water (n=10). Teeth were subjected to microhardness testing before and after artificial demineralization and after remineralization treatment within the four groups.

Results: Following treatment with different solutions in both study and control groups, there was an increase in microhardness after remineralization but with varying degrees. The highest increase in microhardness was shown after remineralization with sodium fluoride followed by agitated Zamzam water.

Conclusion: Zamzam water with agitation causes an increase in the microhardness of the enamel surface after demineralization. Zamzam water is an effective remineralizing agent in initial carious lesions, and its efficacy is comparable to that of sodium fluoride.

## Introduction

Dental caries is one of the most common oral health problems, particularly among children and young adults [1,2]. The primary prevention of dental caries focuses on limiting cariogenic diets, and dental plaque control through individual and professional oral hygiene measures, as well as increasing tooth resistance to acid attack [3]. Increasing tooth resistance to acid attack can be achieved by fluoride which gives hardness and durability to the enamel surface and protects it from caries [4-6]. Water is one of the most essential dietary elements, and its consistency has a significant impact on human health. The use of Zamzam water is very popular [7]. Many Muslims believe that the water of the Zamzam Well is divinely blessed, able to satisfy both hunger and thirst, and cure illness. Pilgrims make efforts to drink this water during their pilgrimage. People living nearby the Zamzam Well might drink the water more than others. The Zamzam Well has been in use for about 4,000 years, according to Arab historians. Zamzam Well is located in the holiest mosque of Muslims, in the city of Makkah. It is about 40 meters deep and surrounded by hills of igneous rocks. For Muslims, drinking water from the Zamzam Well has a unique meaning. Every year, millions of Muslims drink this water as sacred water, especially during pilgrimages and Umrah [8]. In 1971, the Ministry of Agriculture and Water Resources sent samples of Zamzam water to European laboratories for analysis, in order to determine the water's potability. According to the findings of the water samples examined by European laboratories, Zamzam water has a unique physique that makes it beneficial water [9]. The amount of calcium, sodium, potassium, and magnesium salts in Zamzam water differs significantly from other water. The percentage of these minerals was slightly higher in Zamzam water. Most importantly, fluoride has an effective germicidal action [10,11]. In Zamzam water, the four toxic elements arsenic (As), cadmium (Cd), lead (Pb), and selenium (Se) have been found below the toxic level of human consumption. Hence, Zamzam water is safe to drink [12]. The study aims to test the effect of agitated and non-agitated Zamzam water on the microhardness of the artificially initiated carious lesion on the buccal enamel surface in comparison to sodium fluoride gel and deionized water.

## Materials and methods

**Figure 1:**
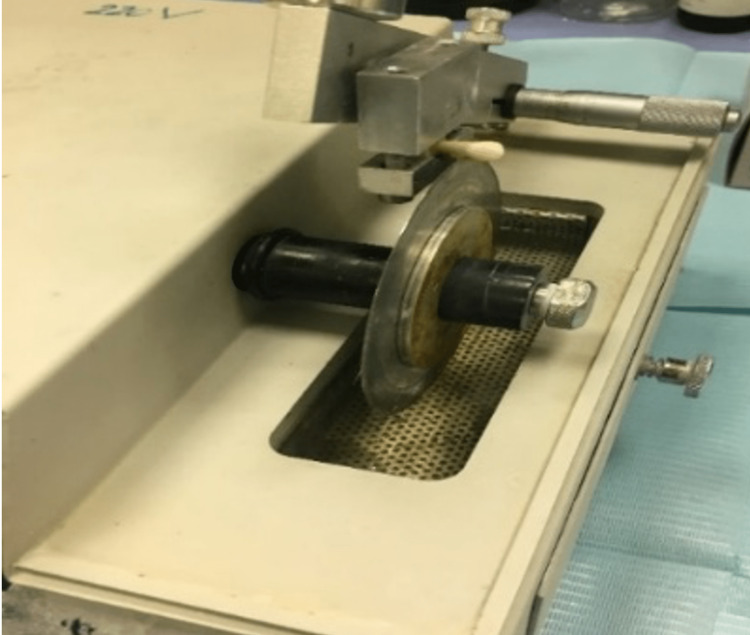
Low-speed saw machine.

This study has been approved by Alrass Dental College, Qassim University IRB (IRB-DRC/12M/4-20) in December 2019. The teeth sample consisted of 40 premolars, extracted for orthodontic reasons. Teeth were washed with deionized water to remove any debris. Teeth were stored in 20 ml deionized water in which 0.1% thymol was added, then kept in 37°C incubators [13]. The total sample (N=40) was divided randomly into study group I (n= 10), study group II (n= 10), positive control group (n=10), and negative control group (n=10). The orthodontic resin was used to encircle each tooth till the cementoenamel junction. After the setting of orthodontic resin, a low-speed saw machine was used to cut the roots of the teeth (Figure 1).

**Figure 2:**
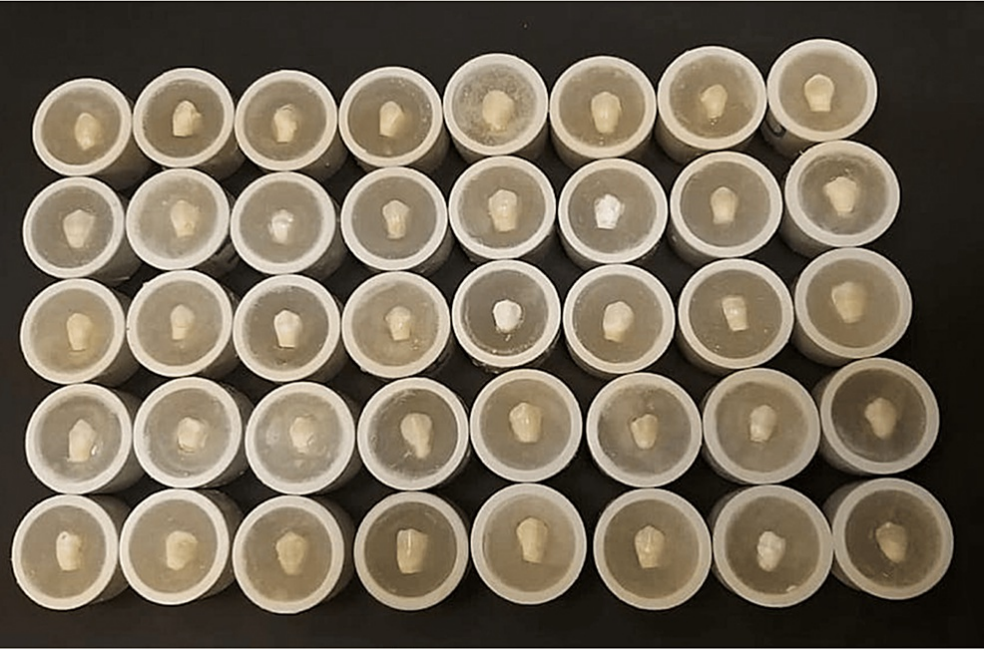
Polyvinyl chloride (PVC) filled with orthodontic resin to hold the crown.

Then, crowns were embedded in polyvinyl chloride (PVC) which was filled with orthodontic resin as well (Figure 2).

**Figure 3:**
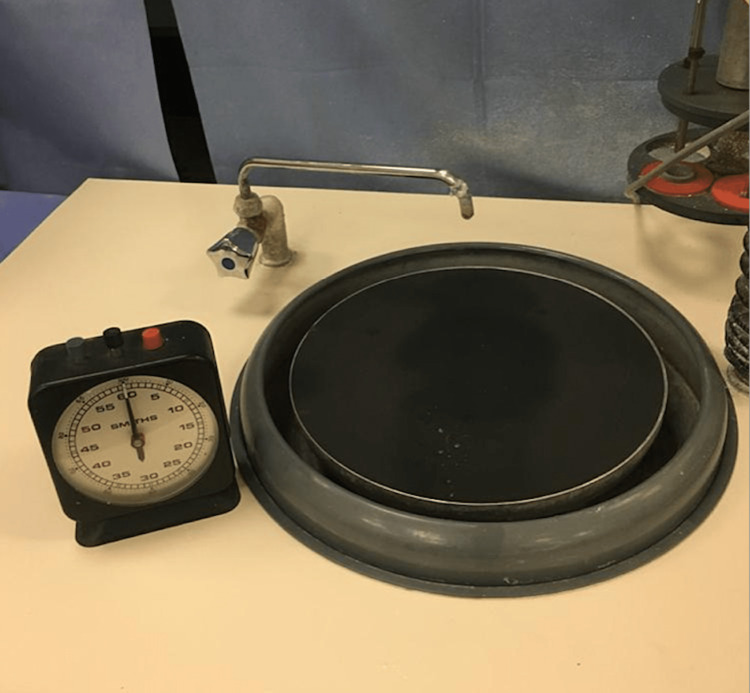
400 grit carbide paper disc in the automatic machine.

Teeth grinding was done using 400 grit carbide paper discs in the automatic machine (Figure 3).

**Figure 4:**
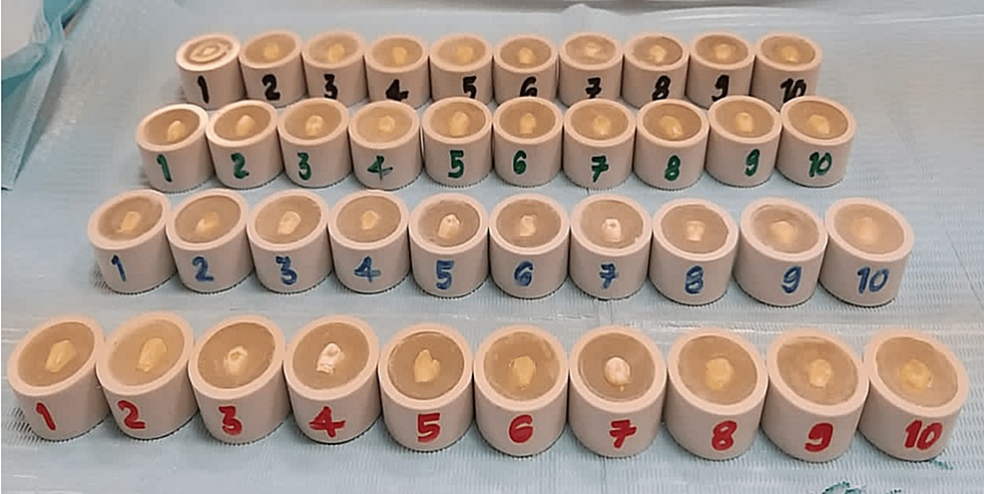
Color coding from one to 10 for each group.

This procedure formed a flat surface on each tooth for microhardness testing [14]. Color coding of the specimens from one to ten in each group was done (Figure 4).

**Figure 5:**
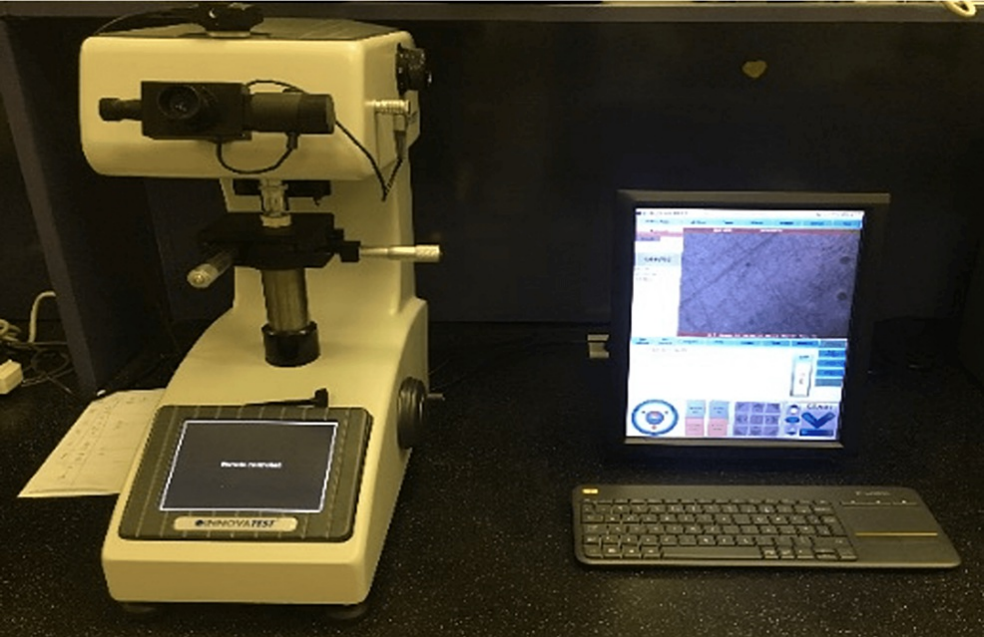
Vickers Microhardness device for microhardness testing.

As baseline reading, all teeth were subjected to microhardness assessment on the buccal surface. Three readings for each tooth were recorded in order to cover as many areas as possible of the buccal surface. The initial microhardness was determined by Vickers Microhardness Device at a load of 500g (Figure 5).

Demineralization protocol to initiate caries-like lesions guided by previous studies with minor modifications [15,16]. The samples were soaked in 20 ml demineralized solution. The potential of Hydrogen (pH) of the solution was adjusted to reach around 4.2 by the addition of potassium hydroxide. The solution was kept at 37°C in the incubator for four days. Then teeth were removed from the demineralized solution and washed with deionized water. After the demineralization process, other readings of the groups were obtained using Vickers microhardness device a at load of 500g.

Each group was treated accordingly: For study group (I), each tooth was immersed in 30 mL of agitated Zamzam water for five minutes. Agitation was performed using a brush. Then treated teeth were rinsed with deionized water for two minutes and placed in deionized water at a 37°C incubator till the next day. This procedure of treating teeth with agitated Zamzam water was repeated daily for two weeks. For study group (II), similar procedure was performed using non-agitated Zamzam water instead of agitated. For control positive group (III), teeth were treated with sodium fluoride gel using the same previous method. For control negative group (IV), teeth were treated as in study groups except that deionized water was used instead of Zamzam water. A third reading for each group was obtained after treatment with agitated Zamzam water, non-agitated Zamzam water, sodium fluoride gel, or deionized water. Microhardness was recorded by Vickers Microhardness device at load of 500g.

Analysis of variance for calculating the significant differences between the different variables was performed. Statistical parameters, mean and standard deviation were calculated. Statistical testing was performed and p-value <0.05 was used as the level of significance.

## Results

Table 1 demonstrates the microhardness mean value and standard deviation of the enamel surface when evaluated at baseline, then after demineralization, then finally after treatment with agitated Zamzam water in the study group (I). The highest mean value of microhardness was recorded after remineralization (388.197 ± 2.13), while the lowest mean value was recorded after demineralization (368.097 ± 4.6).

**Table 1:** Comparison of microhardness values at baseline, demineralization, and remineralization using agitated Zamzam water.

	n	Minimum	Maximum	Mean	Std. Deviation
	Baseline	10	366.8	379.0	372.683	4.2862
Demineralized	10	360.2	376.0	368.097	4.5951
Remineralized	10	385.4	392.3	388.197	2.1325

Table 2 shows the mean value and standard deviation of the enamel surface microhardness at baseline, after demineralization, and after non-agitated Zamzam water treatment in the study group (II). The highest mean value of microhardness was recorded after remineralization (378.473 ± 1.52), while the lowest mean value was recorded after demineralization (367.887 ± 1.23).

**Table 2:** Comparison of microhardness values at baseline, demineralization, and remineralization using non-agitated Zamzam water.

	n	Minimum	Maximum	Mean	Std. Deviation
	Baseline	10	369.7	372.1	370.877	0.9755
Demineralized	10	366.1	369.8	367.887	1.2295
Remineralized	10	375.9	380.4	378.473	1.5185

Table 3 reflects the microhardness mean value and standard deviation before and after demineralization and following sodium fluoride treatment in the control positive group (III). The highest mean value of microhardness was recorded after remineralization (389.620 ± 1.95), while the lowest mean value was recorded after demineralization (372.240 ± 2.30).

**Table 3:** Comparison of microhardness values at baseline, demineralization, and remineralization using sodium fluoride.

	n	Minimum	Maximum	Mean	Std. Deviation
	Baseline	10	374.3	379.1	376.813	1.7065
Demineralized	10	367.5	375.0	372.240	2.3000
Remineralized	10	385.9	392.3	389.620	1.9539

Table 4 demonstrates the microhardness of the enamel surface at baseline, after demineralization, and after treatment. The microhardness increased after remineralization with deionized water in control negative group (IV). The highest mean value of microhardness was recorded after remineralization (379.453 ± 2.82), while the lowest mean value was recorded after demineralization (368.373 ± 6.10).

**Table 4:** Comparison of microhardness values at baseline, demineralization, and remineralization using deionized water.

	n	Minimum	Maximum	Mean	Std. Deviation
	Baseline	10	368.9	374.1	371.693	1.4106
Demineralized	10	358.9	377.1	368.373	6.0980
Remineralized	10	375.5	383.0	379.453	2.8195

**Figure 6:**
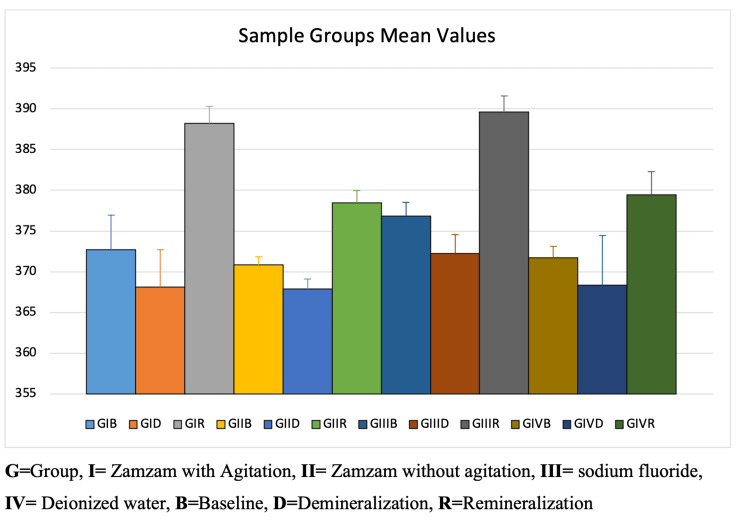
The microhardness mean values of different groups.

Figure 6 shows that following treatment with different solutions in both study and control groups, there was an increase in microhardness after remineralization but with varying degrees. The highest increase in microhardness was shown after remineralization with sodium fluoride followed by agitated Zamzam water. 

Table 5 reflects the mean difference of the remineralized sample of different groups. There is a significant increase (p-value <0.05) in the microhardness of teeth treated with agitated Zamzam water compared to non-agitated Zamzam water and deionized water. Similarly, teeth treated with sodium fluoride showed a significant increase in microhardness compared to non-agitated Zamzam water and deionized water (p-value <0.05). When comparing the two groups there was no statistically significant difference between agitated Zamzam water and sodium fluoride treatment with a p-value of 0.587.

**Table 5:** Comparison between a remineralized sample of each group using one-way analysis of variance.

			Mean Difference	Std. Error	Sig.	95% Confidence Interval	
						Lower Bound	Upper Bound
	Agitated Zamzam water (GI)	GII	9.72333^*^	0.82785	0.000	7.2468	12.1999
		GIII	-1.42333	0.91462	0.587	-4.1266	1.2800
		GIV	8.74333^*^	1.11791	0.000	5.4124	12.0742
	Non-agitated Zamzam water (GII)	GI	-9.72333^*^	0.82785	0.000	-12.1999	-7.2468
		GIII	-11.14667^*^	0.78254	0.000	-13.4746	-8.8187
		GIV	-0.98000	1.01270	0.924	-4.0835	2.1235
	Sodium Fluoride (GIII)	GI	1.42333	0.91462	0.587	-1.2800	4.1266
		GII	11.14667^*^	0.78254	0.000	8.8187	13.4746
		GIV	10.16667^*^	1.08478	0.000	6.9150	13.4183
	Deionized water (GIV)	GI	-8.74333^*^	1.11791	0.000	-12.0742	-5.4124
		GII	0.98000	1.01270	0.924	-2.1235	4.0835
		GIII	-10.16667^*^	1.08478	0.000	-13.4183	-6.9150
*The mean difference is significant at the 0.05 level.							

## Discussion

The primary prevention of dental caries includes increasing the outer enamel surface resistance to acid dissolution and enhancing remineralization. Fluoride is commonly used for the prevention of dental caries since the 1930s [17]. Experimental studies reported that sodium fluoride is successful in remineralizing initial carious lesions and resisting carious attacks [18-20]. As a result, sodium fluoride was used as the positive control in this study. On the other hand, deionized water was used as a negative control.

In this study, the microhardness of the sound enamel surface was recorded from the buccal surface because it has a higher microhardness value compared to other surfaces. This is due to the differences in the mineral composition of the surfaces [21]. A study found that the differences in the mineral composition were due to variations in crystal orientation between the buccal and lingual surfaces [22]. Accordingly, the buccal surface was chosen to be measured in this study.

After soaking the teeth in the demineralized solution, there was a significant reduction in the microhardness of the enamel surface in all groups. This is an indication of enamel surface demineralization. However, an increase in microhardness was observed after the application of sodium fluoride gel, which was statistically significant (p-value <0.005). Another study reported that remineralization of the initial carious lesion occurs as a result of fluoride ions' interaction with the hydroxyl appetite crystals. As a result, a new crystalline substance that differs from the fluorapatite will be formed [23]. Fluoride has been reported to react with hydroxyapatite crystals not only on the surface layer but also on the subsurface layers, giving it a crushing strength greater than the original demineralized material [24]. This explains the rise in microhardness values in teeth treated with sodium fluoride gel observed in this study.

In addition, agitated Zamzam water was effective in rising the microhardness value of demineralized surfaces. As mentioned in a previous study, the incorporation of Zamzam water elements (fluoride, magnesium, calcium) in appetite crystals helps in increasing acid dissolution resistance [10]. The end result of the chemical reaction is not well understood; however, the presence of fluoride components in Zamzam water is responsible for the chemical reaction between Zamzam water constituents and appetite crystals [8].

One of the limitations of this study is the number of samples in each group. In addition, teeth were treated for five minutes up to 14 days only to stimulate using Zamzam water as a remineralization agent in the oral cavity. However, increasing the duration of treatment rather than two weeks could increase the microhardness values. Further studies are needed to confirm this.

## Conclusions

In this study, an increase in microhardness after remineralization was noted in both the study and control groups when treated with different solutions. Among the study groups, the highest mean value of microhardness was recorded after treatment with agitated Zamzam water. Therefore, Zamzam water is an effective remineralizing agent in initial carious lesions and its efficacy is comparable to that of sodium fluoride. However, instructing patients to use Zamzam water as a remineralizing agent in dental practice requires further research. Although this study has statistically proven the remineralization ability of Zamzam water, further studies are needed to confirm the exact scientific mechanism involved in the remineralization process.
